# A case report: primary amoebic meningoencephalitis in a young Zambian adult

**DOI:** 10.1186/s12879-017-2638-8

**Published:** 2017-08-01

**Authors:** Mashina Chomba, Luchenga A. Mucheleng’anga, Sombo Fwoloshi, Joseph Ngulube, Mable M. Mutengo

**Affiliations:** 1 Department of Internal Medicine, University Teaching Hospital, Lusaka, Zambia; 2 Department of Pathology and Microbiology, University Teaching Hospital and University of Zambia- School of Medicine, Lusaka, Zambia

**Keywords:** Primary amoebic meningoencephalitis, *Naegleria fowleri*, Zambia

## Abstract

**Background:**

Primary amoebic meningoencephalitis (PAM) is a fulminant disease of the brain caused by *Naegleria fowleri*. Although the disease is rare, the case fatality rate is very high. In this report, we describe the first case of PAM in Zambia.

**Case presentation:**

The patient presented with sudden onset of seizures and fever on admission. On physical examination he was febrile, comatose and with a stiff neck. Cerebral spinal fluid (CSF) collected on admission did not reveal any organism on microscopy or culture but showed elevated white cell count. A working diagnosis of severe septicemia with acute meningoencephalitis was then made and the patient was started on IV Cephtriaxone (2 g) twice daily. Despite receiving treatment, his condition deteriorated. A second CSF sample collected on day 3 was also negative for bacteria and other organisms. However, a repeat CSF sample collected on day 8 revealed numerous motile organisms that were identified as Naegleria on microscopy and confirmed to be *N. fowleri* on polymerase chain reaction. The patient died on day 8 of hospital admission after having received one dose of Amphotericin B (50 mg). Features consistent with PAM were detected on autopsy.

**Conclusion:**

The isolation of *N. fowleri* in this patient calls for increased awareness among clinical and laboratory staff on suspected PAM cases to promptly diagnose and effectively manage the disease.

**Electronic supplementary material:**

The online version of this article (doi:10.1186/s12879-017-2638-8) contains supplementary material, which is available to authorized users.

## Background

*Naegleria fowleri* is a pathogenic free living thermophilic amoeba mainly found in fresh water bodies such as lakes, hot springs, ponds and recreational spas [1]. *N fowleri* causes primary amoebic meningoencephalitis (PAM), a fulminant disease affecting the brain [2]. The amoeba also known as “brain eating amoeba” enters the olfactory nerve and migrates to the brain through the cribriform form plate [3]. Majority of the infections are acquired during swimming or diving in fresh water [1, 4], although few infections acquired through nasal irrigation and use of piped household water have been documented [5].

PAM is a rare disease with current estimates of recorded cases worldwide standing at just less than 300 [6]. Due to rapid disease progression and delayed treatment in most cases, the fatality rate of PAM is very high with about 95% of those infected succumbing to the infection. If not promptly treated, death occurs within a week of acquiring the infection [2]. Unlike other free living amoeba such as *Acanthamoeba* and *Balamunthia* that cause disease mainly in immunocompromised people, majority of *N. fowleri* PAM cases are observed in young immunocompetent individuals [2]. In this report, we present and discuss the first recorded case of PAM due to *N. fowleri* in a young Zambian male patient.

## Case presentation

**Fig. 1:**
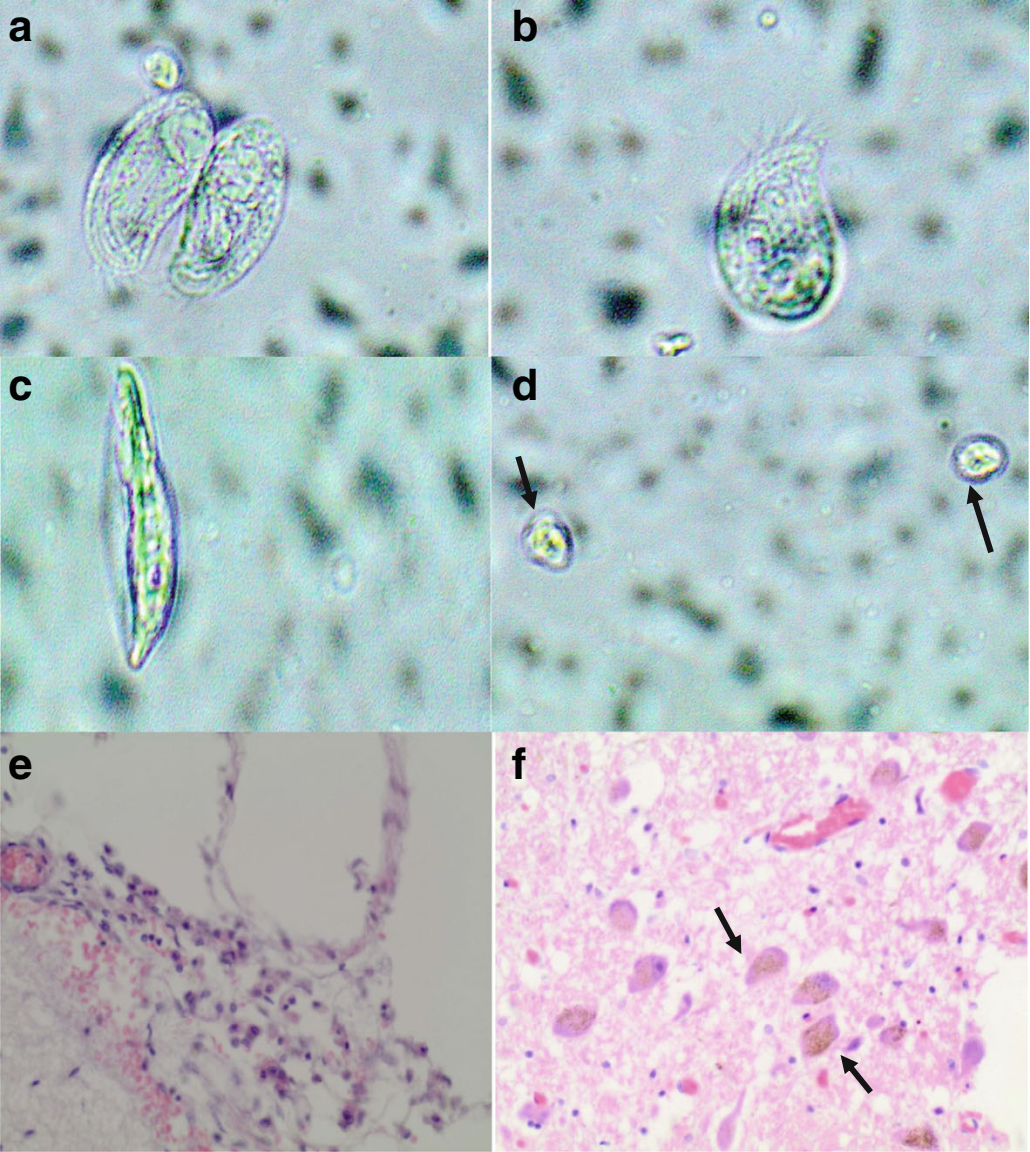
Different stages of *N. fowleri* in cerebral spinal fluid of a 24 year old Zambian male Different forms of Amoeba (**a**, **b**, **c**) and and cysts (**d**) in CSF. Fibrino-purulent material on the leptomeninges, consisting of lymphocytes, neutrophils and congested blood vessels (**e**) and Multiple *N. fowleri* amoeba (**f**) present in the brain parenchyma with hemosiderin pigment

**Fig. 2:**
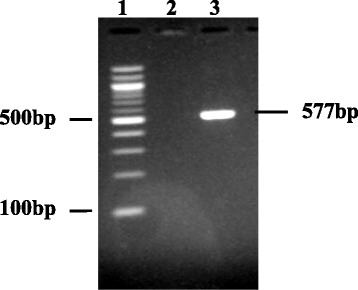
Gel documentation of *Naegleria fowleri* from the Zambia patient’s CSF. Lane 1: Molecular marker. Lane 2: Negative control and Lane 3 showing a 577 bp product of the internal transcribed spacer region of the rRNA *N. fowleri* gene

A 24 year old male police recruit from a training camp in Kafue district, in the Southern part of Lusaka Province was admitted to the University Teaching Hospital in Lusaka, Zambia. He presented with sudden-onset seizures and fever for 1 day with no prior history of ill health. Two days before presenting to our hospital, he was reported to have gone swimming in the Kafue River. On physical examination, the patient was febrile, anicteric and comatose with a Glasgow Coma Scale of 6 out of 15. He also presented with a stiff neck and decorticate posturing. His blood pressure was 104/60 with a pulse rate of 126 bpm and body temperature of 39 °C. Chest and abdominal examination were unremarkable. A rapid diagnostic test for falciparum malaria was negative. There were no features of chronic illness and no palpable lymph nodes were observed. A working diagnosis of severe septicemia with acute meningoencephalitis was made and the patient was started on IV Cephtriaxone (2 g) twice daily. The patient was immediately transferred to the intensive care ward. No bacterial or fungal pathogens were detected in CSF collected on admission (Day 1). Due to poor response to antibiotics, a repeat CSF sample was collected on the third day of treatment but no organisms were detected on direct microscopy or stained preparations. A computerized tomography (CT) scan of the brain did not show any abnormalities. The patient continued to have high fevers with generalized seizures and depression of consciousness, requiring endotracheal intubation and ventilatory support. A direct wet mount microscopic examination of the CSF sample collected on day 8 showed numerous highly motile amoebic trophozoites and cysts (Fig. 1a–d) which was later confirmed to be the pathogenic *N. fowleri* on polymerase chain reaction (Fig. 2). The patient was started on 50 mg of Amphotericin B IV but no improvement was noted. Despite several resuscitations, he died on the same day treatment was commenced (Day 8). Laboratory findings for this case are shown in Table 1 and Table 2. A summary of the patient management is shown in the timeline (Additional file 1).

**Table 1:** Blood Investigations performed on the day of admission (Day 1)

Investigations	Laboratory Finding
Total White blood cell count	16.51 × 10^9^/L
Neutrophils	86.4%
Lymphocytes	5.2%
Monocytes	6.3%
Eosinophils	1.9%
Basophils	0.2%
Random blood sugar	2.99 mmols/L
Malaria Test	No malaria parasites seen
Other blood parasites	No Trypanosomes seen
HIV Test	Negative
Blood culture	No growth detected

**Table 2:** Cerebral spinal fluid findings for Day 1, Day 3 and Day 8 specimens

Cerebrospinal Fluid	Day 1^a^	Day 3	Day 8
White cell count	1120/ mm^3^ (92% neutrophils)	70/mm^3^(82% neutrophils)	200/mm^3^ (90% neutrophils)
Red blood cells	5250/mm^3^	820 /mm^3^	5000/mm^3^
Gram Stain	No organisms seen	No organisms seen	No organisms seen
Indian Ink	No Cryptococcus Spp. seen	No Cryptococcus Spp. seen	No Cryptococcus Spp. seen
Cryptococcal Antigen Test	Non-Reactive	Non-Reactive	Non-Reactive
CSF Culture	No growth	No growth	No growth
Direct CSF Microscopic Examination	No organisms seen	No organisms seen	Amoeba present

^a^Day 1: Day of patient admission to the hospital

Glucose and protein levels were not measured on all the three CSF samples

On Autopsy, the leptomeninges were thin and transparent with vascular congestion, fibrinopurulent exudates (Fig. 1e). There was no displacement of the cingulate gyrus, medial temporal lobe, or cerebellar tonsils. Histology showed edema, lymphocytes and neutrophils features consistent with meningitis. Multiple amoeba were present in the brain parenchyma (Fig. 1f).

To confirm that PAM was due to *N. fowleri*, DNA was amplified with primers NF-ITS-F1 [5′-GAC TTC ATT CGT TCT TGT AGA-3′] and NF-ITSR1 [5′-CTC TTG CGA GGT CCA GAC-3′] that targeted a 577 bp region of the internal transcribed spacer of the rRNA gene [7].

## Discussion

The PAM case described in this paper is the first to be reported in Zambia. This case was detected during one of the hot months with spiking temperatures supporting observations that *N. fowleri* infections are associated with hot seasonality [8]. As has been reported elsewhere, the diagnosis of PAM possesses challenges in that its clinical presentation is similar to that of bacterial meningitis. Due to its rapid progression, death usually occurs within 6–17 days of initial exposure if not promptly diagnosed [9].

The lack of information on PAM in Zambia indicates that there is limited or no awareness among clinical and laboratory staff leading to misdiagnosis of cases. In the few cases of PAM successfully managed [10, 11] in other regions, correct and prompt diagnosis was done and treatment commenced without delay. In the absence of a detailed history of exposure, it is very difficult to clinically diagnose PAM as the infection presents like other types of meningitis. Our case presented with clinical signs and symptoms similar to those that have been previously documented [7, 12]. Symptoms such as severe headache, high grade fever, photophobia, lethargy, confusion with altered levels of consciousness and seizures are commonly observed in patients with PAM. Death in most of the cases is due to increased intracranial pressure [7].

Laboratory findings in PAM patients are consistent and are mostly characterized with increased leucocytes which are predominantly polymorphonuclear cells. CSF may also be purulent with marked leucocytosis, increased protein and reduced glucose levels [7, 12]. In most cases, neuro-imaging investigations in early stages of the disease do not reveal any brain abnormalities [13]. However, a strong suspicion of PAM with history of water exposure should guide the management of patients with such presentations in the absence of bacteria or fungi in CSF specimens.

Critical to survival of PAM patients is prompt detection and aggressive treatment. Currently, the drug of choice is Amphotericin B, an antifungal agent with very low cure rate especially when used as a single drug [14]. The sensitivity of Amphotericin B on *N. fowleri* was demonstrated as far back as the late 1960’s [15]. Other drugs given alongside Amphotericin B are Rifampicin and Fluconazole. In cases where treatment has been successful, aggressive therapy with a combination of antibacterial and antifungal agents was given [8, 16]. Coupled with the use of the above mentioned drugs, management of intracranial pressure, inflammation and induced mild-moderate hypothermia (32 °C-34 °C) are key to successful recovery [16].

Even though the parasite is present in almost all the continents [6, 7], very few cases of PAM have been recorded since it was discovered in 1962. According to the centre for disease control and prevention (CDC), only 138 cases of PAM in the United States have been reported from 1962 to 2015. Recently, there has been an increase in the number of reported PAM cases in Asian countries [14, 17, 18] perhaps due to increased awareness.

In Africa, however, less than 10 cases have been recorded despite having weather conditions such as high temperature that favor the propagation of the parasite. The first reported case of PAM was in an 8 month old baby in Zaria state, Nigeria with no prior exposure to swimming [19]. Following this case, another PAM patient was reported 2 years later in the same country [20]. In addition, there is documentation of *N. fowleri* isolation from environmental sources [21–23]. *N. fowleri* has also been isolated from nasal passages during the dry, windy and dusty months (hamarrtan season) affecting parts of the West African coast [23]. While most of the cases in Africa are from Nigeria, only one case has been documented in Southern Africa [24]. The only case of suspected amoebic meningoencephalitis in a Zambian male gardener was reported in 1974 [25]. In this case, the authors concluded that the infection was not as a result of *Naegleria* but rather another free living amoeba belonging to the hartmannellid family which has been classified as Acanthamoeba. It is conceivable therefore, to suggest that PAM cases are high in Africa and go undiagnosed due to lack of awareness among clinical and laboratory staff.

## Conclusion

This is the first confirmed case of PAM in Zambia. Our case highlights the difficulty of making correct diagnosis when clinical features are suggestive of bacterial meningitis. However, the absence of organisms on gram stain, the lack of response to antibiotics and a recent history of swimming exposure should alert clinicians to the possibility of a diagnosis of PAM. In addition, there is need to raise awareness in both clinical and laboratory personnel on the importance of prompt diagnosis and effective management of patients with suspected PAM.

## Additional file

Additional file 1: Case report timeline. (DOC 60 kb)

## Abbreviations

CSF: Cerebral spinal fluid
HIV: Human immunodeficiency virus
PAM: Primary amoebic meningoencephalitis
