# Supplementary material for: A case report: primary amoebic meningoencephalitis in a young Zambian adult
Source: BMC Infect Dis. 2017 Aug 1;17:532. doi: 10.1186/s12879-017-2638-8 (PMC5540533; doi:10.1186/s12879-017-2638-8)
Supplement: Additional file 1: — Case report timeline. (DOC 60 kb) [file 12879_2017_2638_MOESM1_ESM.doc]

**Male 24 years old, HIV negative. Previously well. Went Swimming in local river 2 days before.**

***28/11/2015***

**Patient Died on day 8**

**New-onset seizures; Fever**

**Semiconscious; Neck Stiffness; BP 104/60mmHg; Pulse 126bpm; Temp 39^o^C**

**Blood Culture, CSF studies (Gram Stain and Biochemistry), Serum Biochemistry**

**Severe Septicemia with Acute Meningoencephalitis**

**Ceftriaxone 2 grams twice daily IV. Moved to ICU**

**Endotracheal Intubation and ventilation**

**Continued to have fever with depression of consciousness**

**Repeat CSF studies ordered on Day 3**

**Trophozoites reported on CSF microscopy**

**Amoebic Meningoencephalitis**

***30/11/2015***

***05/12/2015***

**Amphotericin B 50 miligrams was administered**
